# Insulated at home, indispensable abroad: positioning India's global health strategy after the 2025 contraction in development assistance

**DOI:** 10.3389/frhs.2026.1903108

**Published:** 2026-07-15

**Authors:** Shyamkumar Sriram

**Affiliations:** Department of Rehabilitation and Health Services, College of Public Affairs and Health Sciences, University of North Texas, Denton, TX, United States

**Keywords:** development assistance for health, generic medicines, global health diplomacy, India, pharmaceutical manufacturing, South–South cooperation, tuberculosis, vaccine security

## Abstract

In 2025, development assistance for health fell by about 21% in a single year, driven largely by a 67% cut in United States financing. India sits in an unusual dual position: its core disease programmes, including tuberculosis treatment, are largely domestically financed and comparatively insulated, while much of the developing world depends on it for affordable medicines and vaccines. Falling donor procurement through the Global Fund, Gavi and other channels threatens demand for India-made commodities, while a heavy dependence on imported ingredients leaves the supply base fragile. This brief treats the shock as both a supply-security risk and a strategic opening, and sets out domestic, manufacturing and diplomatic actions to protect access and assume greater leadership.

## Introduction

The financing that underwrote disease control across the developing world contracted abruptly in 2025. After the dissolution of the United States Agency for International Development (USAID) and the cancellation of most of its global health awards, development assistance for health fell by roughly 21% in a single year, a 67% drop in United States contributions, worth more than US$9 billion, accounting for most of the decline (1). Other donors followed. The United Kingdom, France and Germany have all cut their official development assistance for the first time in decades, deepening the shock into 2026 (2). The projected human cost is severe: one modelling study estimated that ending United States funding without replacement would cause roughly 4.1 million additional AIDS-related deaths (uncertainty range 1.6–6.6 million) and about 607,000 additional tuberculosis deaths (95% uncertainty interval 466,000–768,800) between 2025 and 2030, alongside millions of additional child deaths from other causes (3). These are scenario projections that assume no replacement of United States funding and carry wide uncertainty, but even their lower bounds describe a serious reversal.

**Figure 1:**
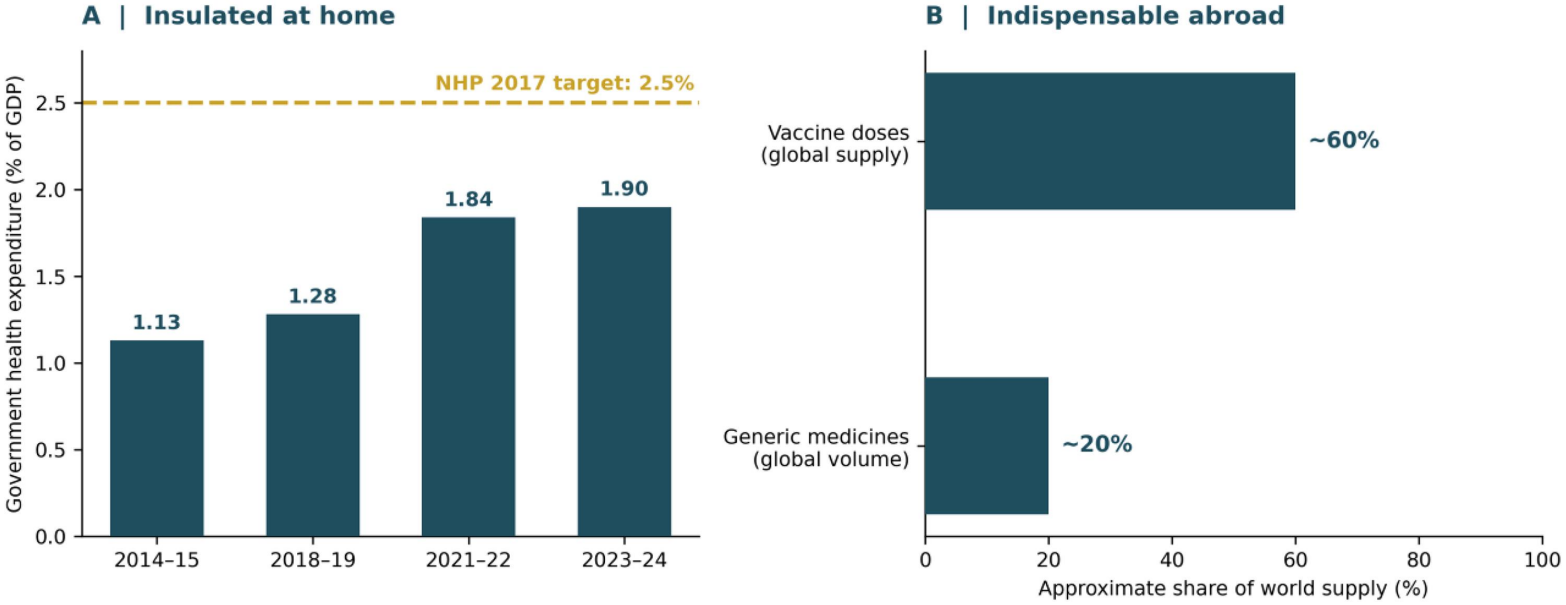
India's dual position in the post-2025 global health order. Panel **A**: government health expenditure as a share of gross domestic product has risen but remains below the National Health Policy 2017 target of 2.5%. Panel **B**: India's approximate share of world supply of generic medicines and vaccine doses. Sources: Economic Survey 2025–26 and National Health Accounts figures (4); Pharmexcil, Indian Pharmaceutical Alliance and UNICEF and WHO procurement data (5–7).

India sits inside this disruption in an unusual way. At home it is largely shielded; abroad it is close to irreplaceable. That combination, set out in Figure 1, is the starting point for this brief.

Consider the domestic picture first. India carries the heaviest tuberculosis burden of any country, accounting for roughly a quarter of the world's cases, yet it pays for most of its national programme itself. The World Health Organization's 2025 tuberculosis report singles India out as the main exception among the United States' priority countries that were otherwise heavily dependent on Global Fund grants (8). On financing, the trend has been upward: the government's share of total health expenditure climbed from 29% to 48% between 2014 and 15 and 2021–22, out-of-pocket spending fell to around 39%, and government health spending now stands at roughly 1.9% of gross domestic product, though this remains short of the 2.5% the National Health Policy 2017 set for 2025 (4). Shielded, then, but not untouched. USAID had put more than US$140 million into the TB Mukt Bharat (TB-Free India) initiative and backed a tuberculosis “buddy” programme reaching some 5.1 million patients across four high-burden states; both were halted in 2025, even as treatment services carried on (9). What the cuts threaten in India is the drive towards elimination, not the core of treatment. The word “shielded” should be read narrowly: it describes treatment, not the whole programme. The cuts have already done measurable harm at the prevention end. The TB-Mukt Bharat financing and the “buddy” support that reached about 5.1 million patients across four high-burden states were withdrawn in 2025, and with them much of the community case-finding, contact tracing and adherence support on which the 2025 to 2030 elimination drive depends. India is therefore shielded on treatment and exposed on elimination, and the harm is not hypothetical but already occurring in the states where these programmes were cut.

The external picture is the mirror image. India is the “pharmacy of the world”, supplying close to a fifth of the global volume of generic medicines and around 60% of the world's vaccine doses; pharmaceutical exports passed US$30 billion in financial year 2025, and Indian medicines reach more than 200 countries (5, 6). The stakes are not abstract. Indian generic antiretrovirals helped bring the annual cost of HIV treatment down from about US$10,000 per patient in 2001 to under US$300, which is what made mass treatment affordable (10). Much of the developing world's supply of artemisinin-based combination therapies, anti-tuberculosis medicines and vaccines, including the commodities purchased through Global Fund and Gavi programmes, passes through Indian manufacturers, which were also among the largest suppliers of COVID-19 vaccines during the pandemic (6).

**Figure 2:**
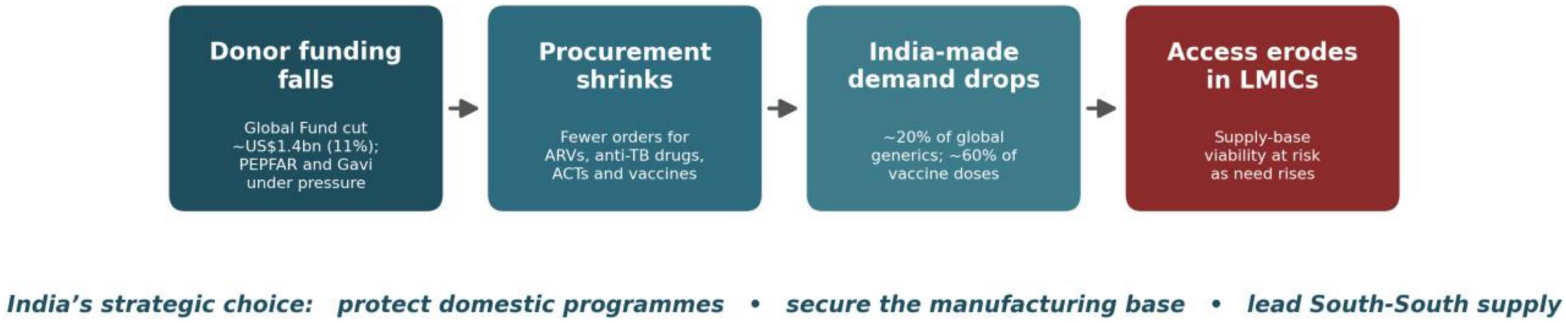
How the 2025 contraction in donor funding transmits to India's medicine supply. Reduced contributions to the Global Fund, Gavi and the President's Emergency Plan for AIDS Relief shrink the procurement budgets that purchase essential commodities, lowering demand for India-made generics and vaccines and putting access across low- and middle-income countries at risk. Sources (6, 8, 12).

These two pictures are connected, and the link is where the danger lies (Figure 2). The money that buys those commodities flows largely through donor channels, and those channels are narrowing. By mid-2025 the Global Fund's 2024–2026 grant cycle had been trimmed by US$1.4 billion, about 11% of its original allocation, with Gavi and the President's Emergency Plan for AIDS Relief under similar strain (8).

These two pictures are not merely connected by narrative; they are linked by the economics of scale. India's price advantage in generics and vaccines rests on very large volumes, and a substantial part of that volume is donor-financed: Indian firms supply the majority of the antiretrovirals used worldwide and close to 60% of the vaccine doses procured through UNICEF (5, 6). When donor procurement contracts, order books for these specific commodities thin out, unit costs rise as fixed costs are spread across fewer units, and product lines that were viable only at scale come under pressure. The same medicines sit at the centre of India's own National AIDS Control and tuberculosis-elimination programmes, so a fall in external demand can raise the price India pays domestically, not only abroad. The feedback runs through employment and investment as well: pharmaceuticals are a major industrial employer, and the bulk-drug Production Linked Incentive scheme alone had created roughly 97,000 jobs by late 2025 (11). A demand shock that idles capacity weakens the very base on which the domestic programmes depend. Stabilising demand, the subject of Option 4, is therefore not only an act of solidarity but a hedge for India's own supply security.

As procurement budgets contract, so do orders for Indian-made medicines, just as need is climbing. A further weakness sits upstream: India imports about 70% of its active pharmaceutical ingredients and key starting materials from China, so its command of finished generics rests on a base it does not fully control (12). The withdrawal of long-standing donors is also leaving a gap in global health leadership that other powers are moving to occupy (1). Precise figures for donor procurement of India-made commodities are not published by supplier country; the estimate here rests on the US$1.4 billion (about 11%) reduction to the Global Fund's 2024 to 2026 cycle and India's majority share of global antiretrovirals and of UNICEF vaccine doses (6, 8).

The argument of this brief is that India should read the 2025 contraction as a risk to supply security and, at the same time, as an opening. What follows sets out a strategy in three parts: protect the domestic programmes, secure the manufacturing base from the ingredient level upward, and turn medicine and vaccine diplomacy into durable leadership of a more self-reliant global health order.

## Policy options and implications

For India the stakes are unusually concrete. It treats the largest tuberculosis caseload in the world, it is the principal supplier of affordable medicines to the Global South, and much of its own upstream supply runs through a single dominant trading partner. The four options below, set out from the most passive to the most ambitious, each address a different part of that exposure. The case made here is that anything short of the fourth leaves one or both halves of India's position vulnerable.

### Option 1

Do little, and treat the cuts as someone else's emergency. Because the core programmes are self-funded, inaction looks defensible in the short run. Over time, though, shrinking donor procurement would hollow out the manufacturing base and erode India's standing, handing influence to rivals already expanding their own global health footprint (1). The insulation is real but temporary, and it would be paid for later.

### Option 2

Safeguard the home front alone. India could ringfence financing for its AIDS and tuberculosis programmes, replace the prevention work stranded by the USAID withdrawal, and push public spending towards the 2.5%-of-GDP mark (4, 9). This is necessary, and it would protect the elimination drive, but it answers only half the problem and leaves India's role as a global supplier to chance.

### Option 3

Protect and strengthen the supply base. The priority here is to scale generic and vaccine manufacturing while reducing the upstream dependence that makes it fragile. The Production Linked Incentive scheme for bulk drugs, worth about ₹6,940 crore (about US$800 million) over 2020–2029, has begun to rebuild domestic capacity for key starting materials and active ingredients, among them penicillin G and clavulanic acid, whose manufacture in India had lapsed for two decades; by late 2025 the scheme had created roughly 56,800 tons of annual capacity (12), although industry observers expect five to seven years before import reliance falls in earnest (13). Alongside this, India can defend the flexibilities it holds under the Agreement on Trade-Related Aspects of Intellectual Property Rights and resist provisions in trade deals that would narrow the generic competition on which poorer countries depend (10). The pay-off is a steadier commodity base for the developing world; the costs are friction with some high-income trade partners and a horizon measured in years rather than months.

### Option 4

Stabilise demand and lead. Building on Vaccine Maitri and a longer record of vaccine diplomacy (14, 15), India could turn *ad hoc* supply into structured partnerships, anchor a Global South pooled-procurement and stockpiling mechanism to steady demand as donor purchasing falls, take a visible part in Global Fund and Gavi replenishment and in carrying out the Lusaka Agenda for Sustainable Health Financing, and offer technology transfer and regional manufacturing, not least in Africa. Pooled procurement is precisely the kind of collective mechanism the Lusaka Agenda promotes to bring down the cost of essential medicines (16), and few countries are better placed than India to anchor one for the Global South. The reward is lasting strategic weight; the conditions are sustained investment, diplomatic effort and a framing built on solidarity rather than commerce.

### Designing the pooled-procurement mechanism

A pooled mechanism will steady demand only if its design answers three questions: who governs it, how it prices, and how it relates to what already exists. Two long-running models mark out the options. The PAHO Revolving Fund has, for more than forty years, pooled the vaccine demand of the Americas under a single price for every member regardless of order size, a solidarity rule that lets the smallest countries buy at the same rate as the largest; it secures savings of around 50%, offers members a line of credit, and PAHO estimates that countries would have paid about 75% more for the region's main routine vaccines outside the Fund (17). The African Pooled Procurement Mechanism, endorsed by the African Union in 2024 and run through Africa CDC, has taken a different route, aggregating continental demand to build local manufacturing as well as to lower price; its first tender, for reproductive and maternal-health commodities, reportedly achieved 30%–90% lower prices than individual-country benchmarks, with about half the awarded products coming from African manufacturers (18).

For India the lesson is not to build a rival platform but to anchor supply within a Global South-owned structure of this kind. Governance should be multilateral rather than India-led, so that partner countries hold the decision rights and India sits as principal supplier and technical partner rather than controller; this is the arrangement most likely to earn trust and to avoid the appearance of a commercial play. Pricing should follow the transparent, cost-plus, single-price logic that has made the PAHO Fund durable, with multi-year framework agreements and firm volume commitments that give Indian manufacturers the demand floor they need to hold capacity. And the mechanism should complement, not duplicate, the Global Fund, Gavi, the PAHO Fund and the African mechanism: India can supply into and aggregate alongside them, adding a demand-aggregation layer that steadies orders as donor purchasing falls. Designed this way, the sovereignty and trust concerns that sink most procurement pools are answered in the structure itself rather than left to goodwill.

None of the first three options is enough by itself. Passivity erodes India's position; defensive safeguarding protects the home front but wastes the wider advantage; and supply-side and leadership measures, though powerful, are slow and demanding. The sensible course pursues all three together, which is how the recommendations below are arranged.

### Who loses and who gains

The contraction does not fall evenly. The prevention and case-finding work that the USAID withdrawal stranded was concentrated on the hardest-to-reach tuberculosis patients: the poor, the undernourished, migrants and informal workers in high-burden states, and the women for whom stigma and limited autonomy already delay diagnosis. The “buddy” support that was cut reached patients who most needed adherence help, so its loss is regressive in its incidence. On the HIV side, prevention cuts bear most heavily on key populations, including sex workers, men who have sex with men, people who inject drugs and migrants, who depend disproportionately on donor-funded outreach. The strategy proposed here is, by contrast, progressive in its benefits: affordable generics and vaccines matter most to the poorest patients in the poorest countries, and a single-price, solidarity-based procurement model of the PAHO type channels its largest gains to the smallest and lowest-income buyers. An equity lens therefore strengthens rather than complicates the case for the Protect, Secure and Lead strategy.

## Actionable recommendations

The actions below fall into three tiers (Table 1), with the supporting evidence drawn together in Table 2. They are meant to run in parallel, because the domestic, manufacturing and diplomatic pressures are arriving at once.

1. **Protect the domestic programmes.** Ringfence and steadily raise domestic financing for the National AIDS Control Program, the National Tuberculosis Elimination Program and routine immunisation, with primary health care first in line and the 2.5%-of-GDP target back in view. The immediate step is to pick up, from domestic budgets, the tuberculosis prevention and community case-finding that the USAID withdrawal cut off, so that the elimination drive does not stall while treatment continues (4, 9). Front-loading this investment, rather than deferring it, protects the progress already made towards elimination and avoids the steeper cost of rebuilding programmes once they have been allowed to lapse.
2. **Secure the manufacturing base.** Deepen self-reliance in active ingredients and key starting materials by sustaining and widening the bulk-drug Production Linked Incentive scheme and the bulk-drug parks, with deliberate support for backward integration into fermentation and chemical intermediates rather than the final steps alone (12, 13). Protect generic and vaccine capacity, defend the country's flexibilities under the Agreement on Trade-Related Aspects of Intellectual Property Rights, hold the line against intellectual-property terms in trade agreements that would curb generic competition, and keep World Health Organization prequalification and quality systems robust so that trust in Indian medicines holds (5, 10).

**Table 1:** Three-tier strategy for India in the post-2025 global health order.

Tier	Objective	Priority actions	Lead actor
Protect	Safeguard domestic disease control	Ringfence and raise NACO, NTEP and immunisation financing; backfill stranded TB-prevention work; move toward 2.5% of GDP	Ministry of Health and Family Welfare
Secure	Protect the affordable-medicines base	Expand bulk-drug PLI and parks with backward integration; preserve TRIPS flexibilities; sustain WHO prequalification	Departments of Pharmaceuticals and Commerce; industry
Lead	Stabilise demand and shape the order	Institutionalise Vaccine Maitri; anchor Global South pooled procurement; engage Global Fund, Gavi and Lusaka Agenda	Ministry of External Affairs with MoHFW

NACO, National AIDS Control Organization; NTEP, National Tuberculosis Elimination Program; TB, tuberculosis; GDP, gross domestic product; PLI, Production Linked Incentive; TRIPS, Agreement on Trade-Related Aspects of Intellectual Property Rights; WHO, World Health Organization; MoHFW, Ministry of Health and Family Welfare.

**Table 2:** Indicators of India's domestic position and global supply footprint. GDP, gross domestic product.

Indicator	Approximate figure	Period/source
Government health expenditure (share of GDP)	1.9%, against a 2.5% target	Recent (4)
Government share of total health expenditure	Rose from 29% to 48%	2014–15 to 2021–22 (4)
Share of global generic medicine supply (by volume)	About 20%	Recent (5, 6)
Share of global vaccine doses	About 60%	Recent (6)
HIV treatment cost enabled by Indian generics	From ∼US$10,000 to <US$300 per patient-year	2001 onward (10)
Active pharmaceutical ingredients imported from China	About 70%	Recent (12)

A strategy that speaks only to government will miss most of the actors who actually make the medicines. India's pharmaceutical base is overwhelmingly private: the Serum Institute of India is the world's largest vaccine manufacturer by volume, Bharat Biotech a major vaccine innovator, Cipla the firm that pioneered sub-dollar-a-day antiretrovirals, and Sun Pharma, Aurobindo and Lupin among the backbone of generic exports, while Laurus Labs and a handful of active-ingredient houses supply the inputs on which the rest depend (7, 11). These firms respond to margins, and the commercial logic now points away from the low-price, donor-funded commodities at the centre of this brief: as donor demand falls, the rational move for a listed company is to shift capacity towards higher-value biosimilars, GLP-1 therapies and regulated United States and European markets, not towards antiretrovirals, paediatric formulations and first-line tuberculosis drugs. That is the central alignment risk. Public policy can close the gap by underwriting demand rather than exhorting firms: a pooled-procurement floor, multi-year volume guarantees, Production Linked Incentive support extended to essential donor-segment commodities, and procurement set-asides can keep the socially important lines commercially viable. Left unaddressed, the misalignment is itself a threat to the “pharmacy of the world”, because that function is sustained by private balance sheets answerable to shareholders, not by public obligation.

(1) **Lead a resilient South–South architecture.** Turn Vaccine Maitri into a standing supply-security partnership with firm volume commitments to low- and middle-income countries; anchor a Global South pooled-procurement and strategic-stockpile mechanism to steady demand as donor purchasing falls; and take an active part in Global Fund and Gavi replenishment and in Lusaka Agenda governance, offering technology transfer and regional manufacturing partnerships, including across Africa (14–16).
(2) **Frame engagement as partnership.** Present all of this as development cooperation grounded in solidarity rather than a commercial play, and pair the supply of commodities with regulatory, surveillance and technical support to partner countries. That combination strengthens access and, with it, India's credibility as a global health leader. The recommendation needs to be honest about the tension it manages. India's pharmaceutical exports are a commercial industry worth more than US$30 billion a year, and the “pharmacy of the world” standing rests on a solidarity narrative; the two are not automatically aligned. The way to hold them together is not to deny the commercial interest but to bind it to development outcomes through visible mechanisms. Four moves help. First, anchor the public story in South–South precedent, from Vaccine Maitri to the antiretroviral price revolution, so that leadership reads as continuity rather than opportunism. Second, pair every supply commitment with non-commercial public goods, including regulatory support, surveillance, technology transfer and regional manufacturing in Africa. Third, adopt transparent, solidarity-based pricing of the PAHO single-price kind, which signals non-extractive intent more convincingly than any communiqué. Fourth, govern the whole through a multilateral structure in which partners hold real decision rights. Managed openly, the commercial-humanitarian tension becomes a source of credibility rather than a vulnerability.

None of this is without cost or risk. Backward integration into active ingredients is capital-intensive and must compete with long-established, lower-cost Chinese production, which is why incentives have so far slowed rather than reversed import growth (13). Defending generic competition may complicate trade negotiations with high-income partners. And a pooled-procurement platform will work only if partner countries trust its governance and pricing. These are reasons to design the measures carefully and to sequence them, not reasons to defer, because the cost of inaction, measured in eroded capacity and lost standing, is the higher one.

### Indicative costs

The recommendations carry very different price tags, and rough orders of magnitude help judge feasibility. Closing the gap between current government health spending of about 1.9% of gross domestic product and the 2.5% National Health Policy target implies additional public outlay on the order of 0.6% of gross domestic product, equivalent to roughly ₹2 lakh crore (about US$23 billion) a year at the current size of the economy, a multi-year commitment rather than a single budget line (4). Backfilling the prevention and case-finding work stranded by the USAID withdrawal is far smaller, on the scale of the US$140 million that the lapsed programmes represented (9). Securing the manufacturing base builds on the bulk-drug Production Linked Incentive scheme already committed at ₹6,940 crore (about US$800 million) over 2020–2030 (12). A pooled-procurement and stockpiling facility would need dedicated seed capital and a revolving working-capital line on the PAHO model, the size of which would depend on the basket of commodities and the number of partner countries (17). These figures are indicative of relative magnitude, not budgeted estimates.

## Conclusions

India comes into the post-2025 order shielded at home and close to indispensable abroad, and neither position will hold on its own. As donors retreat, the demand that keeps the affordable medicines ecosystem viable is shrinking, while a fragile dependence on imported ingredients and a widening gap in global health leadership sharpen the risk. To sit back would protect India's own programmes while squandering its standing as the world's pharmacy. The alternative is active: defend the disease-control programmes and the prevention work the cuts left stranded, build the manufacturing base upward from the ingredient level, and convert medicine and vaccine diplomacy into structured leadership of the Global South. Handled that way, India can hold access steady for hundreds of millions of people and help shape a global health system that leans less on the goodwill of distant donors. The case is as much strategic as humanitarian, and the moment to act is now.
